# Health and Mindfulness during the Syndemic of SARS-CoV-2: An Ethnographic Study

**DOI:** 10.3390/healthcare10040686

**Published:** 2022-04-06

**Authors:** Javier Eloy Martínez Guirao, Fina Antón Hurtado, Fulgencio Sánchez Vera, Anastasia Tellez Infantes

**Affiliations:** 1 Departamento de Ciencia Política, Antropología Social y Hacienda Pública, Universidad de Murcia, 30100 Murcia, Spain; j.eloymartinez@um.es (J.E.M.G.); fmanton@um.es (F.A.H.); 2 Departamento de Sociología y Antropología, Universidad de La Laguna, 38200 San Cristóbal de la Laguna, Spain; 3 Departamento de Ciencia Sociales y Humanas, Universidad Miguel Hernández, 03202 Elche, Spain; atellez@umh.es

**Keywords:** social anthropology, syndemic, health, mindfulness, meditation

## Abstract

SARS-CoV-2 (COVID-19) has caused physical and mental health problems for a large part of the population. In this context, the practice of mindfulness has become relevant due to its usefulness in channeling and mitigating these problems. The objective of this article is to know the implications of the practice of mindfulness in physical and mental health in this syndemic period throh the perception of its practitioners. To achieve this purpose, we conducted an ethnographic study with fieldwork in three mindfulness training and practice groups. The techniques used were participant observation, open interview and survey. Participant observation was carried out from October 2019 to November 2021. A total of 22 open interviews were conducted. In addition, 44 surveys were carried out on a total population of 54. From the first sessions, 45.5% stated that they had felt beneficial effects on their health, and 100% perceived benefits in different aspects of their mental and physical health. The techniques perceived as most effective were those that focused on the body and emotions. The usefulness of mindfulness in managing the problems derived from the syndemic was unquestionable for 95.4%. In addition, a strong correlation is observed between the time spent practicing mindfulness and the reduction in the impact of the syndemic on the health of the practitioners.

## 1. Introduction

The “syndemic” situation [1] caused by the COVID-19 requires a new analysis model in which the biocultural epistemological approach would have to be overcome and the biopsychosociocultural approach should be adopted [2], as it is much more fertile for the analysis of the multidimensional reality in which the current syndemic has placed us. This neologism made up of the term “synergy” and that of “pandemic” aims to broaden the analytical framework of the Western biomedical approach to the disease in a double meaning. The synergy shows that the whole is more than the sum of the parts, which transferred to the health field shows that the coincidence of two or more pathologies supposes a greater aggravation of the patient than they would cause if they had not coincided, which is also increased by the social and environmental conditions in which one lives. The term pandemic refers to the geographical extension of the epidemic, which would affect several countries and in the case of SARS-CoV-2, due to our civilizing process, affects all of humanity. Consequently, the syndemic concept also incorporates social and environmental, political, economic and cultural factors.

It seems that the most fruitful approach would consist of deploying a new paradigm, and here, recovering the words of Krishnamurti [3], we consider that “we can only get out of this crisis through a radical transformation of the mind” (p. 114). This new emerging culture is based on holism, an anthropological term [4] that implies a change in the model of cognition and the interpretation of reality.

### The Syndemic in Spain

This syndemic has been a challenge for 21st century societies and the health of its citizens. On the one hand, COVID-19 has caused a surge in the number of patients admitted to hospitals that has caused many of them to collapse. In Spain, where this investigation has been carried out, they officially registered, according to government sources, more than five million infections [5], and more than 430,000 have required hospitalization, forcing all kinds of consultations and interventions to be delayed. This is a fact that leads to the worsening of the health of people affected by other diseases. Along with this, those who managed to overcome the COVID-19 infection have sometimes had to face long periods of recovery and the possible consequences that this disease has left on their body and mind. On the other hand, the confinement situation has radically affected the management of the “chronotope complex”, understood as “the union of spatial and temporal elements in an intelligible and concrete whole” [6] (p. 259), because as corporeal beings we occupy a space and live for a specific time. In this line, the implementation of restrictive measures that affect mobility (space) and activity (time) generate discomfort. The death of loved ones supposes an emotional loss that is aggravated by the restrictions to celebrate funerals, increasing the suffering of mourning by not being able to carry out the rite, an aspect of special relevance if we consider the anthropological definition of “man” as “Homo ritualis” [7]. The successive so-called “waves of COVID-19” recurrently update the most atavistic fear, as Bauman states: “the original fear is the fear of death, it is an innate and endemic fear that all human beings share, so it seems with the rest of the animals, due to the survival instinct programmed in the course of evolution in all animal species” [8] (p. 46).

This syndemic situation has aggravated the mental health problems typical of the risk society [9]. Recent studies [10] establish that disorders such as depression have increased in the world by 28% during this pandemic, and anxiety disorders by 26%, finding a correlation between the incidence of the virus in each country and the increase in these types of diseases. Faced with this situation of vital collapse, mindfulness is another way to preserve and improve health.

## 2. Related Work

### 2.1. Mindfulness: Holistic Vision of Health, Body and Mind-Body

Mindfulness is a term that was introduced in the West in 1979 by Jon Kabat-Zinn through the Mindfulness Based Stress Reduction (MBSR) program with the aim of reducing stress and chronic pain in patients at the University of Massachusetts Medical Center [11]. The approach was based on mind-body interactions, emphasizing its applicability in the field of health, since “suffering is not an objective condition in the outside world. It is a mental reaction generated by my own mind” [12] (p. 340).

In a simplified way we can understand Mindfulness as the opposite of running on autopilot. However, the term is currently used with two different meanings: on the one hand, it would refer to a state or characteristic of the human mind, present in all individuals to a greater or lesser degree; and on the other, to the psychological technique that allows the development of this state of mind [13]. Regarding the definition of mindfulness as a mental state, the most widespread definition is that provided by Kabat-Zinn: “the awareness that emerges through paying attention on purpose, in the present moment, and nonjudgmentally to the unfolding of experience moment by moment” [14] (p. 4).

Mindfulness does not mean meditation, as it is mistakenly thought. In fact, you can achieve mindfulness without meditating, and not all types of meditation develop mindfulness. Mindfulness is developed through psychological techniques that mainly include attentional meditation [15] (p. 18). Other meditation techniques such as generative or deconstructive techniques develop other qualities of the mind but do not specifically develop a mindfulness state [13].

Although some of the techniques used come from Eastern religious traditions, specifically Buddhism, mindfulness is presented as a secular and scientifically based technique. The first mindfulness protocol, with which it emerged as a therapy, was MBSR (Mindfulness-Based Stress Reduction) [16]. Subsequently, the MBCT (Mindfulness-Based Cognitive Therapy) was developed by Segal et al. [16,17], and others have followed such as MBRP (Mindfulness-Based Relapse Prevention for addictive behaviors) [18]; MBEC (Mindfulness-Based Elder Care) [19] and MBCP (Mindfulness-Based Childbirth and Parenting) [20]. Beyond these standardized and validated protocols, a multitude of Mindfulness-Based Interventions (MBI) have emerged that do not follow the strict criteria of the most frequent protocols in their structure and number of sessions [21]. MBIs are adaptations to diverse contexts, culturally different and oriented to specific populations. MBI should include the formal basic techniques of the MBSR and MBCT protocols [13]. These techniques can be divided into essential and accessory. Essential would be: mindfulness in breathing, body scan, and the three-minute practice; and the accessory ones would be mindfulness walking, mindfulness in body movements, looking only by looking and listening only by listening, and interpersonal mindfulness [22]. This system maintains a vision of health and the body that differs from that held by hegemonic medicine in Western countries.

Mindfulness tries to distance itself from the vision of conventional medicine that tends to treat the different systems and organs of the body in a separate and decontextualized way. Biomedicine does not recognize the importance of the interaction between person and environment as an expression of the tension that is established between the subject and the context. We have a corporeity that forces us to occupy a space and it modifies us. It is, therefore, a bidirectional influence. According to Cognitive Anthropology, and more specifically Ecological Cognitivism [23,24,25,26], the environment in which we develop our lives conditions and shapes us, focusing on multiple processes of ontogenesis [27] that are present through the practice of mindfulness. It is, therefore, in line with different traditional and alternative medicines that maintain a holistic vision of health, and to a certain extent, more in line with the WHO’s own definition, which considers health as a “complete state of well-being: physical, mental and social” [28].

This vision is shared in different cultural traditions around the world. For example, as Flores Guerrero [29] recounts, in numerous Ibero-American indigenous cultures the health system is understood as the harmonious functioning of the physical, mental and spiritual aspects of a person: in the Kichwa of Ecuador, health is the achievement of harmony and balance of the physical, the mental and the spiritual in the individual, the community, the culture, nature and the earth, which allows human development in the biological, social and spiritual aspects [30]; for the Aymara people of Bolivia, the closest concept to health would be “kankaña” which means well-being, moral peace and physical integrity [31,32]; in the Shipibo-Conibo and Asháninca of Peru it is the state of normality and balance between human being/spirit, human being/family, human being/social group and human being/nature [33]; in the Ngöbe of Panama it is the product of a harmonious relationship with the environment, with human beings, with nature and with the gods [34].

In the same way, Chinese medicine, which shares principles with Taoism (also with Buddhism and Confucianism), conceives health as a state of spiritual, mental and physical harmony with nature. The human body is considered as an integral whole and its different parts are interrelated and must function in harmony [35,36,37]. Likewise, one of the fundamental principles of the *dao* or path that these belief systems must be followed is to live in harmony with nature [38,39,40,41]. Mindfulness, derived from Buddhist meditation practices, maintains that holistic view.

### 2.2. Scientific Evidence and Configuration of the Imaginary

In the last decade, there have been numerous studies on the beneficial effects of mindfulness on health. These investigations have concluded that it is effective in treating disorders such as depression [42], as it favors the development of a prosocial attitude [43], improves anxiety management [44,45,46], blood pressure, cortisol levels and other physiological markers of stress [47], addictions [48], and attention deficit disorders [49,50], among others. But beyond the clinical application, the practice of mindfulness is considered beneficial for the “healthy” population, since, as pointed out by Tang, Hölzel and Posner [11], it improves the self-regulation of attention, emotions and self-awareness.

Self-regulation can be understood as the voluntary management of executive functions [51] so that an individual can define their own goals, establish plans, initiate activities and regulate the tasks and skills necessary to be able to carry them out efficiently. More specifically, self-regulation refers to the ability of a subject to manage their thoughts, actions, emotions, motivation, and direct their learning [52]. The cognitive processes involved are attentional flexibility, (focusing attention, changing perspective and adapting flexibly to changes; working memory (retaining and processing information); and impulse control (inhibiting automatic or impulsive responses to achieve a goal). The proper development of these functions allows the subject to manage actions aimed at an objective and to give adaptive responses to new or complex situations [53]. Different studies support that the greater capacity for self-regulation is correlated with greater physical health and well-being [54], greater longevity [55], and the reduction of addictions, such as tobacco [56], etc.

Likewise, studies indicate that meditation techniques (such as practices for regulating focused attention on a chosen object, or open monitoring attention, that is, nonreactive monitoring of the content of experience from moment to moment) produces a decrease in negative mood states and anxiety [57,58], and an improvement in positive mood states [59].

On the other hand, the practice generates a more positive self-representation, a higher self-esteem and those who practice it have a greater acceptance of themselves [60,61]. In addition, over time, mindfulness practice becomes a trait. Scientific research on long-time meditators shows changes in areas of the brain related to stress and anxiety [62]. Specifically, the prefrontal cortex, the cingulate cortex, and the hippocampus show increased activity, and the amygdala shows decreased activity consistent with better emotional regulation.

Studies of mindfulness practitioners who performed standardized eight-week programs, such as MBSR, also showed brain changes similar to those in traditional meditation practice [63]. Consequently, authors such as Behan [64] have pointed out that “the introduction of a practice of mindfulness and meditation during this pandemic has the potential to complement treatment and is a beneficial low-cost method of providing anxiety support for all”. Since the predominant symptoms that occur in patients and in society are anxiety, feeling overwhelmed and despair, this is where mindfulness has shown good results. In this sense, its usefulness for people who work in the health service has been suggested [65], since it reduces emotional fatigue and encourages the development of tools for emotional regulation, helping to prevent mental health-related problems [66].

Although there is still not much research on this, it would be logical to think that “strict health protocols, in particular containment measures, may not be detrimental to mental health when one transforms his experience of anxious loneliness into an avenue of meditation practice as a mental health self-care strategy” [67].

### 2.3. The Rise of Mindfulness during the Syndemic

**Figure 1:**
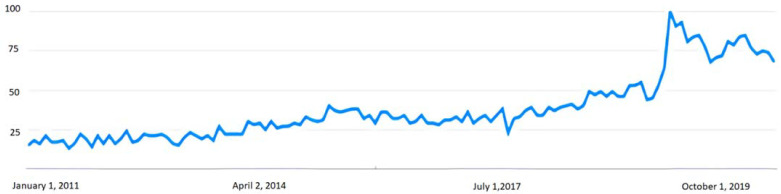
Number of searches for the term “meditation” between 2011 and 2021 in the West. Source: Google Trend.

The offer and practice of meditation and mindfulness linked to its effects on health has been expanding in Western countries in recent years. This process has accelerated during the current pandemic. As we can see in Figure 1, the number of searches for the term “meditation” on Google doubled at the beginning of the quarantines and lockdowns, reaching its maximum in the first week of April 2020, after which it has remained 25% above the average of previous years. This fact shows how meditation aroused the interest of the population as a way of self-care.

**Figure 2:**
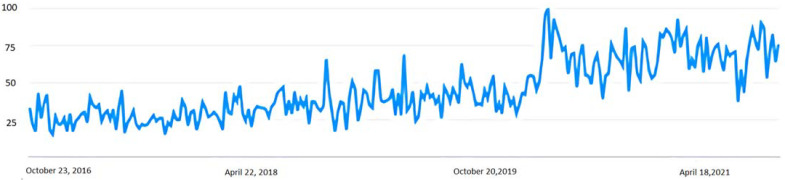
Number of searches for the term “meditation” between 2016 and 2021 in Spain. Source: Google Trend.

In the case of Spain, the trend has been similar. In Figure 2, we can see how interest in this topic soared with the arrival of the pandemic and how this interest has remained high after confinement. Under this demand, many centers that carried out mindfulness activities redirected their activity to cyberspace.

## 3. Purpose of the Work

The objective of this article is to analyze the effects of the practice of mindfulness, according to the perception of its practitioners, in the maintenance and improvement of mental and physical health during the current syndemic period. This objective is divided into five specific ones: to analyze the health impairment due to the syndemic, to determine the motivation for the practice of mindfulness, to specify the degree of improvement in health perceived by the practitioners, to explore the applied techniques and their effectiveness in health; and, finally, to examine the role that mindfulness has had in coping with the syndemic and in the resilience of professionals. To answer these questions, we have carried out an ethnographic study with fieldwork in three mindfulness training and practice groups from October 2019 to November 2021.

## 4. Materials and Methods

This article is part of a broader investigation that tries to study several groups of MBI practitioners in Spain. The results that we present in this study are based on a case study in a mindfulness and transpersonal development center. The training center is run by a certified instructor with twelve years of experience in mindfulness group training. Initially, the center was located in Murcia (Spain), and due to the syndemic it moved its activity to cyberspace.

### 4.1. Participants and Procedures

The training courses run from October to May. The center usually welcomes about thirty students per course, between new and continuing. Specifically, in the 2019–2020 academic year there were 30 students; in 2020–2021 there 32, of which 13 were new and the rest came from the previous year; and in the current academic year there are 32 students, of which 11 are new students. In total, 54 students have been trained at the center in this period (N = 54).

The research made use of qualitative techniques such as participant observation and open interview; and quantitative techniques such as the survey. Through the qualitative techniques of participant observation and open interview, it was possible to access detailed information and understand the meanings of the participants. On the one hand, the participant observation allowed us to capture what the practitioners did in their training contexts, while the open interviews gave us access to the discourses that were generated around the practice. On the other hand, the quantitative technique of the surveys allowed us to determine the representativeness of the responses. The application of the different techniques allowed us to triangulate the results, that is, to compare and contrast the results produced by each research technique [68,69].

The participant observation within the group was carried out between October 2019 and November 2021. The classes were delivered in person and later online. A total of 22 open interviews were conducted, 21 with students and one with the instructor. In addition, 44 student surveys were conducted out of a total population of 54.

The informants’ selection criteria for the 44 students who have been surveyed were experience in meditation practice, age, and sex.

### 4.2. Sociodemographic Characteristics of the Informants

As shown in Table 1, all of our informants are between 35 and 67 years old, which are the youngest and oldest ages found in the people who are members of this group. Specifically, they are distributed as follows: 22 informants were between 35 and 45 years old, 14 were between 46 and 55 years old, and eight were over 56 years old. Regarding sex, the majority, 38, are women, compared to a total of 6 men.

In terms of years of experience, four were inexperienced, with less than a year of experience; 12 had between one and two years of experience; eight had between two and three years, six had between three and four years of experience, six had between four and five years, four had between five and six years, two had between six and seven, and two had more than seven years of experience.

## 5. Results

To show the results we are going to divide them into five sections. In the first section, we will show the data related to the deterioration of health due to syndemics. In the second, we will investigate the motivation for the practice of mindfulness. The third section will be dedicated to addressing the relationship between mindfulness and health improvement. Later, in the fourth section, we will analyze the effectiveness of the different techniques used in practice, and, in the last section, we study the effect of mindfulness in improving the main health problems caused indirectly by the syndemic.

### 5.1. Health Impairment Due to Syndemic

**Figure 3:**
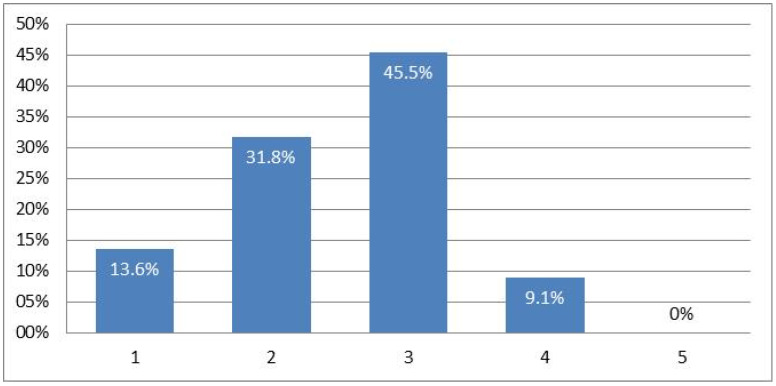
Degree of affectation at the mental level.

As we have pointed out, the syndemic has had important consequences on the health of the Spanish population. In this sense, 86.4% of our informants stated that it had affected their mental health in some way. When asked to what degree the mean scored 2.5 on a scale of 1 to 5, where 1 is not at all, 5 is a lot, and 3 is the midpoint (see Figure 3).

The informants alluded to problems generated by anxiety, stress, and depression caused by loneliness, physical distancing, lack of contact with family and friends, loss of social relationships, their job, their loved ones, and the uncertainty of the situation.

“My social relationships have diminished, it has negatively affected my state of mind, my fears and insecurities have increased, it has removed me emotionally, and it has made me worse and better”.

(male student, 40 years old, six years of experience)

“Well, I ended up with my self-esteem on the ground and with a lot of fear of what surrounds me”.

(female student, 46 years old, one year of experience)

It is significant that 14.3% stated that the pandemic had not affected them on a mental level. As they commented in the qualitative interviews, these informants perceived it as an opportunity for introspection, to connect with nature and alluded to the values of mindfulness when facing it:

“I could say that this time of introspection and being alone with myself has been good for me”.

(female student, 44 years old, five years of experience)

“It has provoked in me greater introspection, more interest in investigating myself and my relationship with my environment”.

(female student, 50 years old, two years of experience)

“My case was different because when we saw that they were going to confine us, we moved to a house that we have in the mountains. So, it can be said that I spent it mostly outside where there was more or less normality, so it affected me little”.

(female student, 43 years old, three years of experience)

“Confinement has not caused me any problems. I am aware that many people have been affected. But I have really handled it well, with acceptance and calm”.

(Female student, 50 years old, five years of experience)

**Figure 4:**
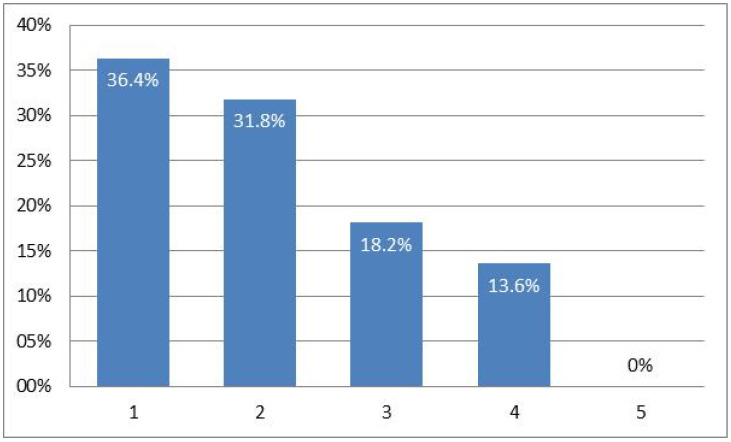
Degree of physical or corporal affectation.

This affectation was also evidenced on a physical level, referred to by 63.6%, although with a perceived average score of 2 (where 1 is not at all, 5 is a lot and 3 is the midpoint) as can be seen in Figure 4.

The types of conditions reported in the qualitative interviews were related to physical inactivity due to confinement, somatizations of the mental state itself, and the overwhelming of the health system that made it impossible to control the usual diseases that arose in time.

### 5.2. Motivation for the Practice of Mindfulness

When we asked them about the motivation to practice mindfulness, the informants mentioned aspects such as relaxation and calm of the mind, anxiety and emotional stress as the main motivations that led them to practice mindfulness. Sometimes it was due to feeling emotional discomfort caused by stressful life circumstances such as separations, problems with work, family health, etc., or due to existential problems.

“I tried it [mindfulness] for the first time in a meditation and transpersonal development workshop, and I continue to practice it until now. It all started with the search to alleviate that discomfort and negativity that consumed me in life at that time”.

(male student, 40 years old, six years of experience)

“Mainly due to emotional discomfort derived from my work. On the other hand, as a way of acquiring tools and being able to apply them at work and with my family”.

(female student, 39 years old, three years of experience)

“What was my main motivation for mindfulness? Well, in my case I resorted to mindfulness because of the need to know who I am”.

(female student, 37 years old, two years of experience)

“I wanted to find my inner peace, because I was a roller coaster. I was absolutely afraid of everything, from the time I got up until I went to bed… I still have, there are times when I get up and say: “oh! I’m like a fear of something and I have to stop!”… Meditation has served me at a brutal level”

(female student, 54 years old, seven years of experience)

As the informants show us through the interviews, mental health acquired an even more relevant place as a motivation for the practice of mindfulness. Furthermore, it is remarkable that all the informants say that somehow they have found what they expected.

### 5.3. Mindfulness and Health Improvement

For all informants, the practice of mindfulness has had some effect on their level of physical, mental or emotional health. Meditation has brought peace into their lives, they say, in addition to self-control, relaxation, serenity, balance, tranquility, acceptance, trust, understanding, etc.

“Greater knowledge of myself and my emotions, knowing and being aware of how I respond to stimuli, the reason for my behaviors in certain cases, tools to better respond to situations, and above all to be able to identify my state. It has also given me more coherence in my day-to-day life”.

(female student, 39 years old, two years of experience)

**Figure 5:**
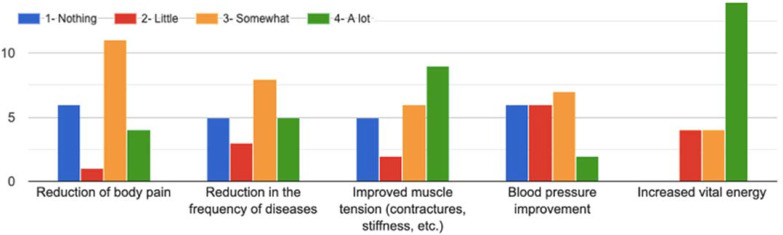
Degree of incidence in the physical health aspects improved by the practice of mindfulness.

All practitioners claim to have noticed physical changes since they performed the meditation, as shown in Table 2 and Figure 5:

The increase in vital energy is what they have perceived the most, and where the informants have been more unanimous. In fact, 63.6% state that it has increased a lot, 18.2% somewhat and 18.2% a little.

The improvement in muscle tension (contractures, stiffness, etc.) is another aspect that is perceived by the majority (77.3%), while 40.9% believe that it has improved a lot, 27.3% somewhat, and 9.1% a little. Only 22.7% say that it hasn’t improved.

There are also the majority (76.2%) who have the perception of a reduction in the severity of their disease due to practicing mindfulness. Specifically, 23.8% say it has reduced a lot, 38.1% somewhat, and 14.3% a little bit.

The reduction in body pain is perceived by 72.7% of the informants. Of them, 18.2% feel that it was reduced by a lot, 50% somewhat and 4.5% a little bit.

Finally, 82.7% also stated that mindfulness had helped them to regulate their tension. Although in this case only somewhat (31.8%) or a little bit (27.3%).

But where the effects of mindfulness are most perceived is with regard to mental health. In the qualitative interviews, our informants mentioned internal aspects such as the reduction of anxiety, the feeling of peace and tranquility, the improvement of self-control, the reduction of negative emotions, the improvement of self-compassion and self-care, and the reduction of depressive states. These internal aspects were projected into external attitudes and behavior that implied an increase in empathy, greater tolerance and greater compassion, which in turn entailed an improvement in social relationships.

“I have gone from crying without knowing why, to being sad, to being stressed, to not being able to sleep, to taking pills, I was taking I don’t know how many pills, the doctor told me: ‘You will never be able to get rid of this treatment’. … And I no longer take depression or sleeping pills, I sleep like a dormouse, I play sports, I feel good…”

(female student, 54 years old, seven years of experience)

“Begin to live in a different way, with awareness of my life experience. A very large rupture of conceptual schemes linked to entrenched emotions, which have been the basis of my life until now. With a new vision, I take responsibility by breaking schemes and creating new ones, thereby beginning to create a new life experience. Begin to feel like owner, responsible and creator of my life”.

(female student, 56 years old, one year of experience)

All these effects were confirmed in the surveys, which showed that all the informants had experienced each of them to some degree.

As can be seen in Table 3, the most perceived aspects are the reduction of anxiety, where 72.7% said they had felt it a lot and 22.7% somewhat, the feeling of peace and tranquility (86.4% a lot and 9.1% a little) and improvement in self-control (77.3% a lot and 18.2% a little). The rest of the aspects are perceived in the majority also to the highest degree (a lot).

**Figure 6:**
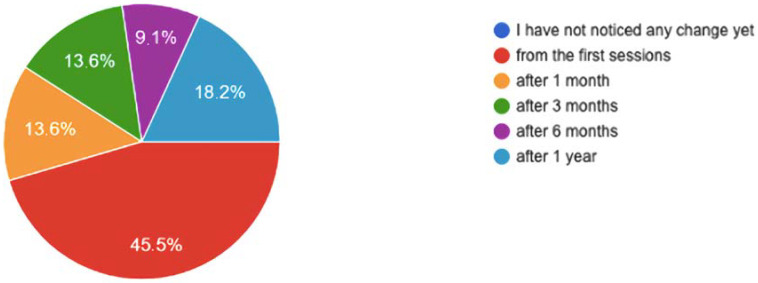
Degree of incidence in the mental health aspects improved by the practice of mindfulness.

As shown in Figure 6, these beneficial health effects were perceived from the first sessions by 45.5%, and only 18.2% took more than a year to become aware of them.

The association between the practice of mindfulness and improved health is a unanimous perception among all informants. In this sense, the instructor tells us about some experiences of radical improvement that she has been able to witness throughout the twelve years as an instructor:

“Very radical changes can occur. My experience with people who have had oncological processes confirms that the mental change in their relationship with the disease is absolutely crucial for following treatment and managing suffering. […] In the case of people with chronic depression who have been taking medication for a long time, I have also seen improvements when they face what happens to them and meditate, I remember a student who, after taking Prozac for five years, was able to stop medication”.

(female instructor, 50 years, 25 years of experience)

Although they perceive its great effectiveness in the field of health, they are also aware of its limitations:

“[Mindfulness] It is aimed at people who may be going through a crisis, but they still have the resources and ability to help themselves, the ability to learn and enough motivation to be constant in the practice. You have to be motivated, because if you find yourself in a deep depression and you don’t find the motivation, it won’t work, it won’t do you any good”.

(female instructor, 50 years old, 25 years of experience)

### 5.4. Mindfulness and the Effectiveness of Techniques

The techniques most practiced and perceived as efficient are those that focus attention on bodily or emotional processes, as illustrated in Table 4. Thus, the technique perceived as most efficient is attention to breathing, which was efficient to some degree for all of the participants, 68.2% who found it to be very efficient and by 27.3% as efficient. Attention to the body (body scan) also stands out, as 96.5% consider it efficient to some degree, 40.9% as very efficient and 45.5% as efficient. The third technique that stands out is attention to emotions, perceived with some degree of efficiency by 91% of the informants, of which 40.9% describe it as very efficient, and also 40.9% as efficient. The techniques least used and perceived as less effective are those that pay attention to external objects such as sounds or images. Thus, half of the informants do not use attention in the images, and only 9.1% rate this strategy as very efficient. Likewise, attention to sounds is not used by 36.4% of the participants, and it is considered very efficient by only 22.7% of those who use it.

### 5.5. Mindfulness and Syndemic

**Figure 7:**
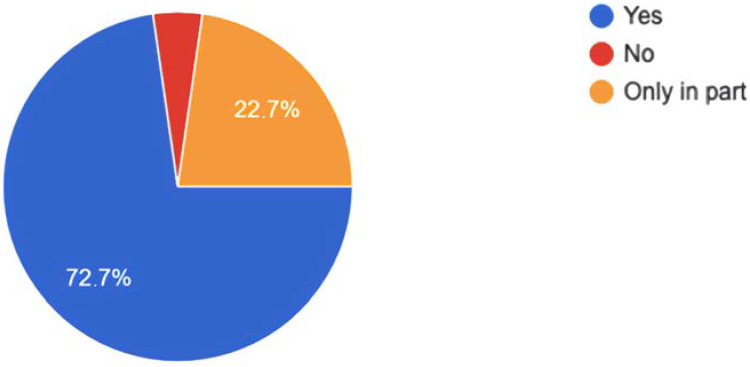
Perception of the usefulness of mindfulness practice during confinement and situations derived from the syndemic.

The efficacy of mindfulness in improving the main health problems caused indirectly by the syndemic seems quite clear. In this sense, the informants have the perception that mindfulness was useful to them in managing the quarantines and lockdowns and other situations that resulted from the syndemic. As reflected in Figure 7, 72.7% state that it has been useful to them, while 22.7% state that it has also been useful, although only partially.

Comparing the time of experience as a meditator and the negative effects of the syndemic perceived by informants on their health, we find that there is a clear correlation. Specifically, the longer the experience in mindfulness, the lower the perceived negative effects on health, especially in those with more than three years of experience.

As reflected in Table 5, considering the values within a scale from 1 to 5, where 3 is the midpoint, in the case of mental health, the informants with less than three years of practice have been affected by an average of 3 points, while for those with more than three years of experience the degree of involvement drops to 1.9 points. The same occurs with physical involvement, as the group with the most experience values it with an average of 1.8 points, compared to 2.25 and 2.2 points for the less experienced practitioners.

## 6. Discussion

In the current syndemic situation [1], the distancing and isolation measures, the rupture and alteration of the space-time structure in daily life [5], the loss of loved ones and the impossibility of celebrating ritual farewell acts [7], the fear of death [8] and illness, the loss of economic and employment stability, the presence of a new invisible “enemy” that threatens one’s existence together with the feeling of risk and uncertainty [9] are all emotions that have been experienced by people. These are feelings that have had negative effects on the health of a large part of the population [10]. The data from this research confirm that the mindfulness practitioners studied have experienced the same circumstances and have been negatively affected by the syndemic. However, the research also shows that the adoption of a different perspective and a relativization of the problems generated by the syndemic through mindfulness has been understood by many as an unprecedented opportunity not only to counteract many of the negative consequences for health but to channel the stress related to the syndemic and the experience of loneliness towards positive growth and greater resilience, confirming the thesis of Deguma et al. [67].

The search for health and its maintenance is an important motivation to start and stay in the practice of mindfulness, according to our informants. Specifically, anxiety and emotional stress were the main factors that led them to practice mindfulness, and relaxation and calmness of the mind was the goal or aspiration to continue with the practice. In short, mental health is the fundamental motivation for the practice, and persistence in it is explained by the fact that all of the participants have perceived benefits in both their mental and physical health, and 45.5% affirm that they have felt beneficial effects in their health since the first practice sessions. These results coincide with other previous studies that relate the practice of mindfulness with the improvement of health [43,44,45,46] and its maintenance [59].

The effects on health are achieved by using a technical repertoire that includes techniques that focus attention on the body, emotions, mind and the external environment. It is precisely the techniques that focus directly on the body and emotions that are practiced the most, and in turn the most accepted and perceived as most effective. The techniques that pay attention to external objects are, on the other hand, the least valued. The explanation for this may be related, in part, to cultural factors that condition the perception of health, inclining it towards the hegemonic biomedical conception in the society under study. This conception of health focuses, on the one hand, on the body, and on the other, on the management of emotions also linked to the proper functioning of the body itself. The ideas of health from a more holistic perspective, which imply harmony with the environment and nature, although they are included in mindfulness-based interventions, are not appreciated with the same intensity in the informants’ practice.

The usefulness of mindfulness in managing the consequences of the syndemic on health is verified. Practitioners have no doubts, since 95.4% agree with this statement. In addition, there is a correlation between the time of practice and the degree of negative impact of the syndemic on health. In the case of practitioners with more than three years of experience, the perception of health deterioration is significantly lower.

Finally, we highlight a significant finding: the majority of mindfulness practitioners we have encountered in our research are middle-aged women. This could be due to a combination of biological and cultural factors. The mind-body relationship is substantially different between men and women, because the neuronal distribution and the endocrine system are different and all of this is due to the predisposition of the woman’s body for motherhood [2]. Gestation is a psychoneuroimmunoendocrinological process that reinforces the mind-body connection, hence the influence that psychological factors have on genetic and epigenetic regulation [70], the integrity of chromosomes, intra and intercellular communication, communication of organs and systems and physiology [71]. The secretion of neurohormones such as prolactin activates the protective instinct, due to our mammalian condition, and enhances self-care practices. This practice is being reinterpreted in Western society by trying to overcome the traditional cultural ascription of gender, which could be summarized in the maxim “take care of yourself to care” and replacing it with “care for personal well-being”. From a sociocultural point of view, it must also be borne in mind that in Western societies, where there is still a sexual division of patriarchal work, women mainly assume the tasks of caring for the domestic group, and in these last two years of the pandemic, they have the overexertion of work and the consequent stress of maintaining the cleaning and prevention guidelines against the virus fell on them. Women have been and are largely responsible for buying the appropriate masks, disinfecting clothing and shopping, attending to the health of the family physically and psychologically, making the place of work compatible during confinement, doing domestic work, teleworking, etc. They, in a great majority, have been subjected to high levels of stress without having time to themselves to decompress, which has prompted them to seek, even more than in pre-pandemic times, gateways like practicing yoga, meditation, or, as we have discussed in our research, mindfulness. As evidenced in a recent study by the Women’s Institute of the Spanish Ministry of Equality in December 2020, the pandemic has shown that “women continue to do most of the domestic work and care for dependent people, paid and unpaid, also assuming a greater mental load derived from it. Furthermore, many women are forced to not be able to continue working due to having to cope with care tasks when schools are closed” [72] (p. 3).

To conclude, we want to indicate that these data and findings must be considered within the limitations of the investigation. It is important to point out that this research follows the perspective of social anthropology and, therefore, it is focused on the in depth study of a specific community at a specific time and context, in our case in a mindfulness training center during the syndemic. Consequently, the results, although they corroborate and extend the results of previous studies, cannot be generalized. In any case, it is necessary to continue expanding the research to other groups, both in similar and different sociocultural contexts, as well as the analysis of the diversity of practices and techniques that are practiced.

## 7. Conclusions

The COVID-19 pandemic has abruptly changed our way of life. Confinement drastically reduced, in Heideggerian terms, our being and being in the world. It prohibited mobility, both social and geographic, which is so valued in 21st century society. It stifled the expression of affections, the basis of our process of hominization and humanization. It inoculated a diffuse but effective fear with the end of death and installed a general feeling of unease. This disturbing situation has generated not only physical but also mental health problems. Our scientific and technological development has made it possible to reduce it through vaccines and information and communication technologies, which have been key in maintaining social relationships, but the current situation requires a holistic and systemic rethinking of our conception of the notion of health. It is necessary to reconnect mind and body to achieve that state of well-being and resilience that is achieved with mindfulness, as has been exposed in this contribution, in which we have given our informants a voice.

Mindfulness has proven to be a useful tool for managing the various health effects of the syndemic. The combination of different practices and techniques performed constantly over a period of time has contributed significantly to the perception of the reduction of both mental and physical conditions generated directly or indirectly by COVID-19. In this way, mindfulness is making an important contribution and can continue to do so in improving public health in syndemic contexts.

## Figures and Tables

**Table 1:** Sociodemographic characteristics of the informants.

Age Range	Number of People	Sex
35–45	22	4
46–55	14	2
56–67	8	0

**Table 2:** Physical health aspects improved by mindfulness practice.

	Nothing	Little	Somewhat	A Lot
Reduction of body pain	27.3%	4.5%	50%	18.2%
Reduction in the frequency of diseases	22.7%	13.6%	36.4%	22.7%
Improved muscle tension (contractures, stiffness, etc.)	22.7%	9.1%	27.3%	40.9%
Blood pressure improvement	27.3%	27.3%	31.8%	9.1%
Increased vital energy	0%	18.2%	18.2%	63.6%

**Table 3:** Aspects of mental health that have been improved by the practice of mindfulness and grade.

	Nothing	Little	Somewhat	A Lot
Anxiety reduction	0.0%	0.0%	22.7%	72.7%
Feeling of peace and tranquility	0.0%	4.5%	9.1%	86.4%
Improved self-control	0.0%	4.5%	18.2%	77.3%
Improved social relationships	0.0%	4.5%	31.8%	63.6%
Decrease in negative emotions (fear, anger)	4.5%	0.0%	36.4%	59.1%
Decrease in negative thoughts	4.5%	0.0%	27.3%	68.2%
Increased empathy	0.0%	0.0%	36.4%	63.6%
Greater tolerance	0.0%	0.0%	31.8%	68.2%
Greater compassion	0.0%	0.0%	36.4%	59.1%
Improved self-compassion and self-care	0.0%	9.1%	27.3%	63.6%
Stress reduction	0.0%	4.5%	45.5%	50.0%
Reduction of depressive states	0.0%	13.6%	36.4%	45.5%

**Table 4:** Efficacy of the techniques practiced.

	I Don’t Use It	Not Efficient at All	Not Very Efficient	Efficient	Very Efficient
Mindfulness of breathing	0.0%	0.0%	4.5%	27.3%	68.2%
Attention to sounds	36.4%	0.0%	9.1%	31.8%	22.7%
Attention in images	50.0%	0.0%	9.1%	27.3%	9.1%
Use of mantras	22.7%	9.1%	9.1%	45.5%	13.6%
Visualizations	22.7%	0.0%	9.1%	40.9%	27.3%
Attention to the body (body scan)	4.5%	0.0%	9.1%	45.5%	40.9%
Attention to emotion	4.5%	4.5%	9.1%	40.9%	40.9%

**Table 5:** Years of experience in mindfulness and perception of the negative effects of the syndemic.

Years of Practice	Mental	Physical
1 or less	3	2.25
between 1 and 3	3	2.2
3 or more	1.9	1.8

## Data Availability

The data presented in this study are available on request from the corresponding author.
